# A novel human IL-2 mutein with minimal systemic toxicity exerts greater antitumor efficacy than wild-type IL-2

**DOI:** 10.1038/s41419-018-1047-2

**Published:** 2018-09-24

**Authors:** Xi Chen, Xiaojie Ai, Chunlian Wu, Heyong Wang, Gang Zeng, Peixin Yang, Gentao Liu

**Affiliations:** 1 Shanghai Biomed-Union Biotechnology Co. Ltd, Shanghai, China; 2 Center for Translational Medicine, Shanghai Pulmonary Hospital, Tongji University School of Medicine, Shanghai, China; 3 Department of Obstetrics, Gynecology and Reproductive Sciences, University of Maryland School of Medicine, Baltimore, MD 21201 USA; 4 Department of Biochemistry and Molecular Biology, University of Maryland School of Medicine, Baltimore, MD 21201 USA; 5 School of Agriculture and Biology, Shanghai Jiaotong University, Shanghai, China; 6 School of Life Science, China West Normal University, Nanchong, Sichuang Province China; 7 Central Laboratory, Shanghai Pulmonary Hospital, School of Medicine, Tongji University, Shanghai, China; 8 Department of Urology, David Geffen School of Medicine, University of California, Los Angeles, CA USA; 9 Department of Oncology, Shanghai Orietal Hospital, Tongji University School of Medicine, Shanghai, China

## Abstract

IL-2 is critical to the activation, growth, and survival of T cells and NK cells, and maintains the delicate balance between auto-immunity and anti-neoplasm surveillance. High IL-2 doses have clear antitumor capabilities, but also have severe side effects that limit its clinical use. Side effects include the vascular leak syndrome (VLS), which results in lung edema and liver damage. Therefore, a new version of IL-2 that does not induce organ toxicity would improve IL-2-based immunotherapy. We conducted a systematic screening by changing one amino acid at a time at the interaction area of IL-2 with its receptor IL-2R to select one particular mutant IL-2, FSD13, in which the proline at position 65 was substituted by lysine (P65L). FSD13 had a greater ability than wild-type IL-2 in stimulating CD4^+^ T, CD8^+^ T, and NK cell proliferation, enhancing the expression of CD69, CD183, CD44, and CD54 in these cells, and triggering cancer cell apoptosis. FSD13 had three-time lower than wild-type IL-2 in inducing CD4^+^ T to Tregs. Compared with wild-type IL-2, FSD13 greatly limited the growth, invasion into adjacent tissues, and metastasis of melanoma metastatic into the lung. In contrast to wild-type IL-2, high dose of FSD3 did not alter structures and induce any pathogenic changes in the liver and lung. Thus, we generated a novel the IL-2 mutant, FSD13, by targeting a different area than previously reported. FSD13 surpasses the wild-type IL-2’s ability in stimulating the antitumor immune cell functions, but exerts much less systemic toxicity.

## Introduction

Interleukin-2 (IL-2), a small (15.5 kDa), four α-helical bundle cytokine, which is mainly produced by CD4^+^ Th1 cells, activates CD8^+^ T cells and natural killer (NK) cells. IL-2 has crucial roles during both the immune system’s resting and activated states^1^. IL-2 receptors (IL-2Rs) consist of three subunits: IL-2Rα (CD25), IL-2Rβ (CD122), and IL-2Rγ (CD132)^2^. IL-2 can bind to CD25 alone, a heterodimer consisting of IL-2Rβ (CD122) and IL-2Rγ, or a heterotrimer consisting of CD25, CD122, and CD132. These three different constructions of IL-2R form low-, intermediate-, and high-affinity IL-2R, respectively. Unlike IL-2Rβ and IL-2Rγ, which meditate signal transportation downstream of IL-2, IL-2Rα only enhances the affinity between IL-2 and IL-2Rs.

Because of IL-2’s therapeutic potential in stimulating proliferation of the main antitumor immunocytes, namely CD8^+^ T cells and NK cells in vitro, it is used in clinical immunotherapy. The use of IL-2 to stimulate an effective immune response against metastatic cancers, such as melanoma and renal cell carcinoma, dates back to the early 1980s. In several clinical trials, high doses of IL-2 led to the regression of advanced cancers in selected patients with metastatic renal cell cancer, melanoma, colorectal cancer, and non-Hodgkin’s lymphoma^3^. Administration of unmodified IL-2, either alone or with antigen-specific treatments, has resulted in remarkable long-term survival of certain patients suffering from metastatic melanoma^4^. However, several clinical trials suggest that only 15–20% of treated patients receive clinical benefit from IL-2^5^. This low success rate is due to two main reasons. First, even low doses of IL-2 induce the proliferation of regulatory/suppressor T cells (Tregs). Tregs are a specialized subpopulation of T cells that suppress the activation, expansion and function of other T cells^6^, thereby dampening antitumor efficacy. Many cancer patients exhibit an increased number of Tregs. In some cases, such as melanoma and ovarian cancer, high numbers of Tregs correlate with a poor prognosis^7^. Second, the widespread use of IL-2 is hampered by dose-dependent adverse effects, such as hypotension, pulmonary edema, liver cell damage, and renal failure^4^. Clinical trials have shown that high-dose IL-2 administration can induce complete tumor regression in a small number of patients, and many patients have experienced extended disease-free intervals^8^. Paradoxically, the high doses of IL-2 required to obtain such results induce high toxicity, with VLS being the most frequent and severe complication^9^.

Strategies in designing IL-2 muteins aim either for the increase of CD122 binding affinity or the decrease of CD25 binding affinity^4^. For the latter, IL-2 muteins have been generated by replacing R38, F42, Y45, and E62 with alanines^2^. These muteins have comparable antitumor efficacy with wild-type IL-2 but possess lower toxicity^2^. In the present study, we substituted twelve individual amino acids between positions 37 and 72 by lysines in designing low-affinity CD25 muteins. We found that a new IL-2 mutant (FSD13) with the P65L substitute exerted significantly higher capability than the wild-type IL-2 in promoting the proliferation of CD8^+^ T cells and NK cells without massively increasing the number of Tregs. Furthermore, in contrast to wild-type IL-2, FSD13 exhibited negligible organ toxicity.

## Results

### FSD13 more effectively stimulates antitumor immune cells than wild-type IL-2

**Fig. 1:**
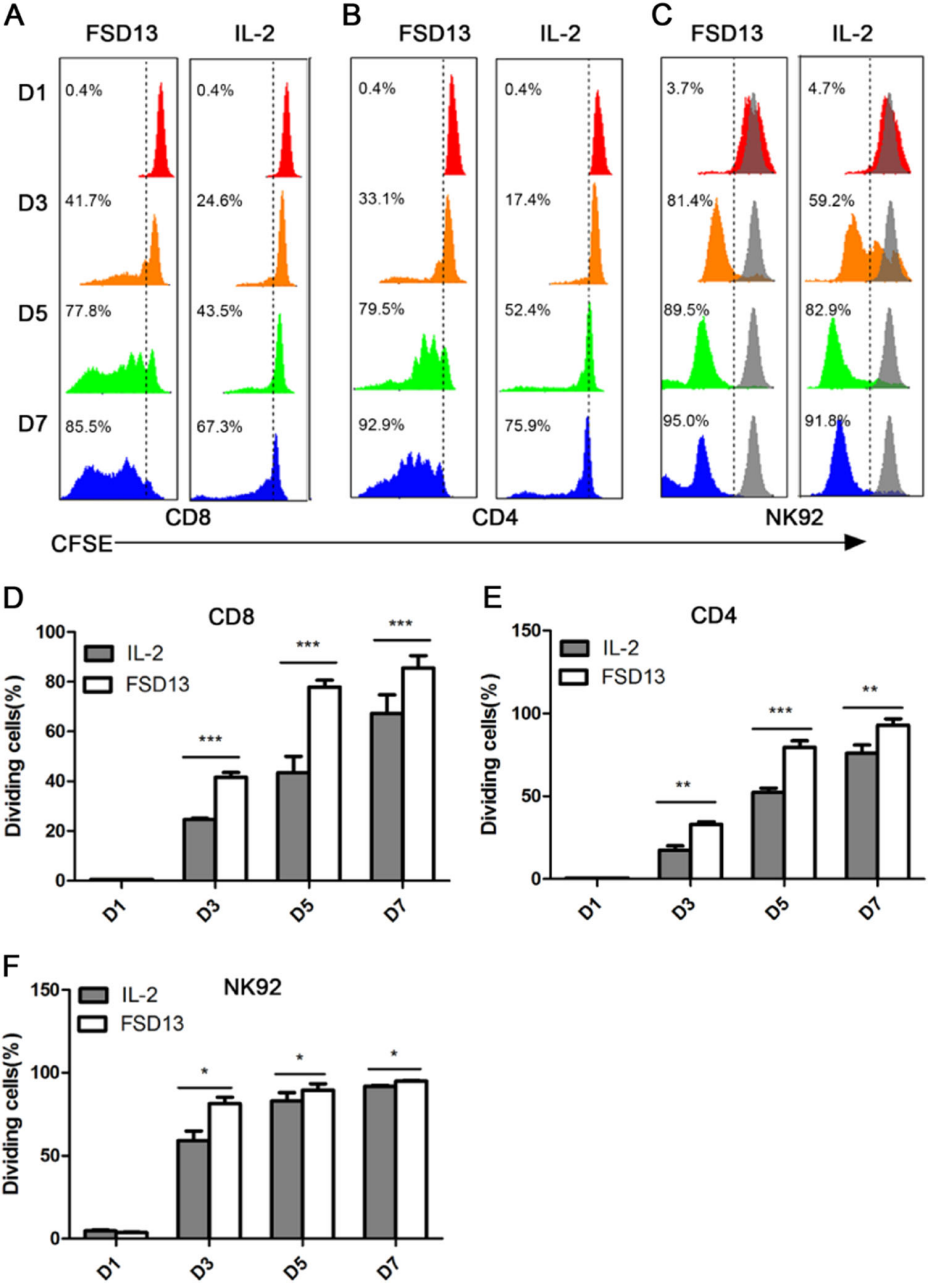
Proliferation Assay of CD8^+^ T cells, CD4^+^ T cells and NK92 Cells. CD8+ T cells and CD4+ T cells were purified from hPBMC. Then, CD8+ T cells, CD4+ T cells, and NK92 cells were labeled with CFSE before administration of FSD13 or IL-2; PBS served as control. All experiments were repeated three times. **a**, **d** FSD13 showed a better ability to stimulate CD8+ T cells to proliferate. **b**, **e** FSD13 improved the proliferation of CD4+ T cells. **c**, **f** FSD13 displayed a stronger promotion effect on the expansion of NK92 cells. All experiments were repeated three times. Experiments were conducted in five biological replicates (*n* = 5) for each group. Data were analyzed by Student’s *t*-test and are represented as mean ± SEM. *P < 0.05, **P < 0.01, ***P < 0.001

Numerous studies have shown that IL-2 signals affect T cells during all stages of an immune response, including primary expansion, contraction, memory generation, and secondary expansion^10^. CD4^+^ and CD8^+^T cells were separated using magnetic separation and labeled with CFSE (5(6)-carboxyfluorescein *N*-hydroxysuccinimidyl ester) before conducting a proliferation assay. We used FSD13 or wild-type IL-2 to stimulate the two subpopulations of T cells for 7 days, to determine whether FSD13 had the same ability as wild-type IL-2 to induce T-cell proliferation. We used fluorescence-activated cell sorting (FACS) on days 1, 3, 5, and 7. On day 1, no significant difference was observed on CD4^+^ and CD8^+^ T cells stimulated by either kind of IL-2. The advantage of FSD13 was gradually seen, first appearing on day 3. Among CD8^+^ T cells, 41.7% of the CD8^+^ T cells stimulated by FSD13 were divided, whereas the dividing ratio for cells stimulated by wild-type IL-2 was only 24.6%. On day 5, the difference in the dividing ratios was even greater (FSD13: 82.2% vs. wild-type IL-2: 40.1%). On day 7, although 90.5% of the CD8^+^ T cells in the FSD13 group were divided, the dividing ratio among cells stimulated by wild-type IL-2 increased to 60.6% (Fig. 1a–d). The same phenomenon was observed in CD4^+^ T cells treated with FSD13 or wild-type IL-2. The difference in the dividing ratio (FSD13 minus wild-type IL-2) appeared on day 3, 15.7% on day 4, 30.4% on day 5, and 23.7% on day 7 (Fig. 1b–e). In a proliferation assay of NK cells, NK92 cells labeled with CFSE were treated with FSD13 or wild-type IL-2 for 7 days. A proliferation assay was performed on days 1, 3, 5, and 7 using FACS. Significant differences in the dividing ratio between the FSD13 and wild-type IL-2 groups began appearing on day 3 (*P* < 0.05). NK92 cells stimulated with FSD13 had an overall advantage in proliferation. On day 3, more than 80% of the NK92 cells treated with FSD13 divided, whereas the dividing percentage of NK92 cells treated with wild-type IL-2 was limited to about 60% (Fig. 1c–f).

### Changes in phenotype on T cells after treatment with FSD13 or wild-type IL-2

**Fig. 2:**
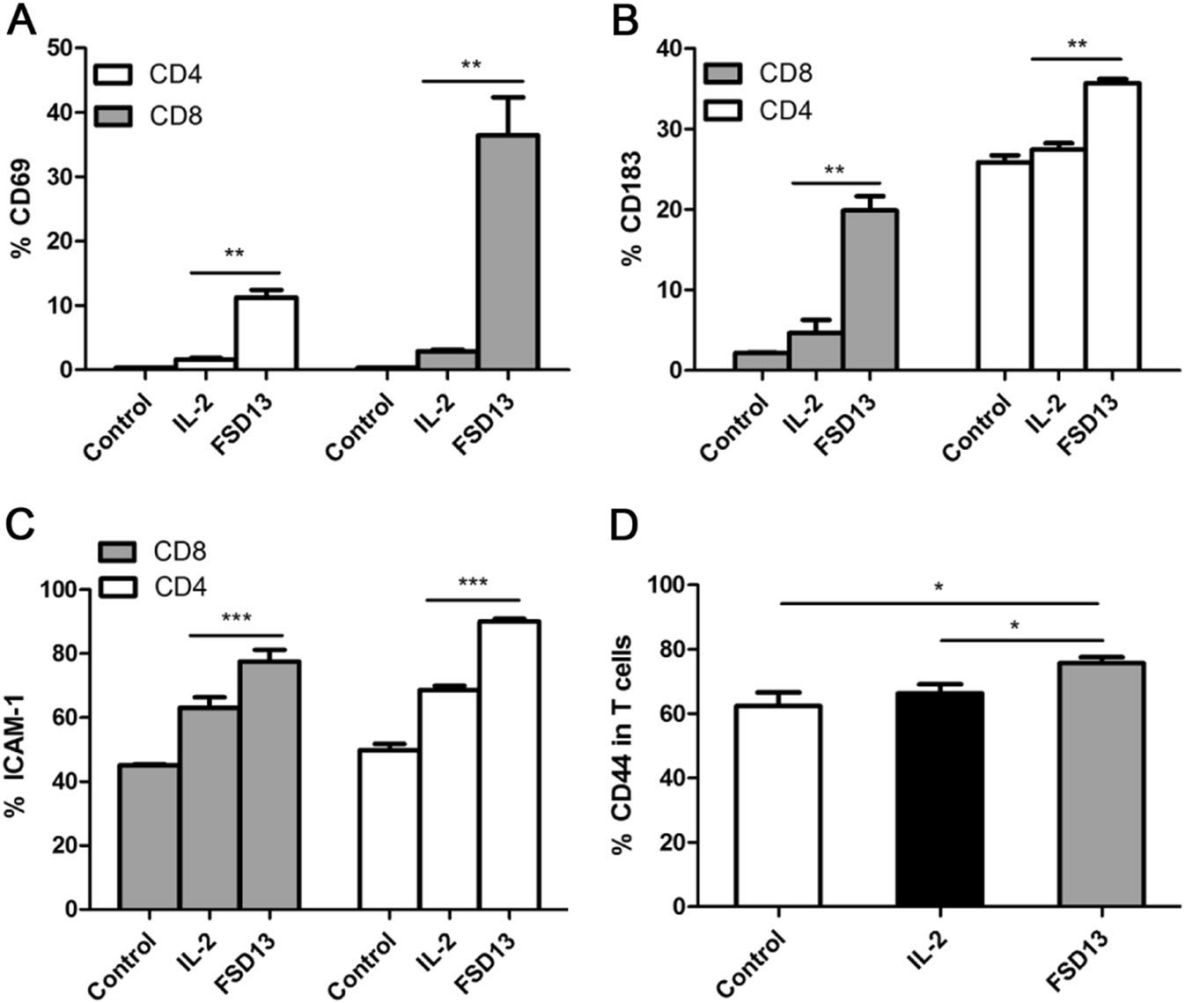
Phenotype changes on T cells after FSD13 or IL-2 treatment. After T cells were purified from hPBMC, they were treated by the some dose of FSD13 or IL-2; PBS served as control. For the assay of CD69 expression, experiments were conducted 24 h after administration of IL-2. Expression of CD183, ICAM-1 and CD44 on T cells after treatment was analyzed on day 5 after administration of IL-2. All experiments were repeated three times. **a** FSD13 increased CD69 expression on T cells. **b** CD183 expressed on T cells was significantly increased after FSD13 treatment. **c** ICAM-1expression on T cells improved after FSD13 administration. **d** CD44 expression was higher on T cells stimulated by FSD13 (*P < 0.05, Student’s *t*-test). All experiments were conducted three times. Experiments were conducted in five biological replicates (*n* = 5) for each group. Data were analyzed by Student’s *t*-test and represented as mean ± SEM, and were collected from three independent experiments. *P < 0.05, **P < 0.01, ***P < 0.001

**Fig. 3:**
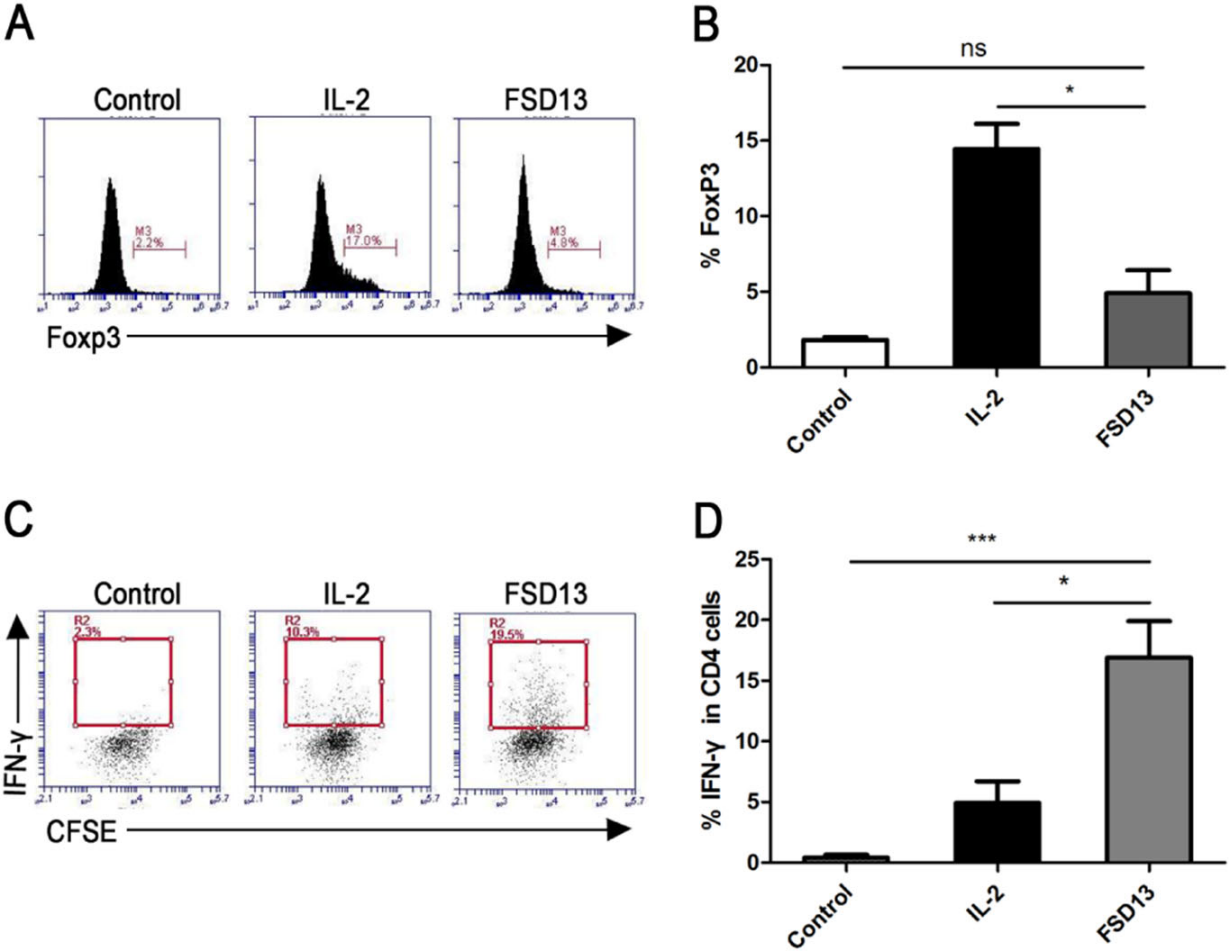
Effect on Tregs and IFN-γ secretion. CD4+ T cells were purified from hPBMC before they were treated by the same doses of FSD13, IL-2 or PBS (control). FACS analysis was performed on day 5 after administration of IL-2. **a**, **b** Compared with IL-2, FSD13 decreased the frequency of Tregs in purified CD4+ T cells. **c**, **d** FSD13 increased IFN-γ secretion in purified CD4+ T cells. All experiments were performed three times. Experiments were conducted in five biological replicates (*n* = 5) for each group. Data are represented as mean ± SEM and were collected from three independent experiments. *P < 0.05 (Student’s *t*-test)

T cells purified from PBMCs (peripheral blood mononuclear cells) were treated with FSD13 or wild-type IL-2 before they were tested for changes in phenotype related to T-cell immigration and activation. During the early stages of T-cell activation, CD69 expressed transiently. Once expressed, CD69 acts as a co-stimulatory molecule for T-cell activation and proliferation^11^. Before FACS analysis, CD4^+^ and CD8^+^ T cells were treated with FSD13 or wild-type IL-2 for 24 h. Compared with T cells treated with wild-type IL-2, the frequency of T cells expressing CD69 was significantly higher in FSD13-treated CD8^+^ and CD4^+^ T cells, presenting almost 19 times more expression in CD8^+^ T cells and a 7-fold increase in CD4^+^ T cells (Fig. 2a). An assay of CD183, CD44, and CD54 expression frequency on activated T cells was conducted on day 5 after treatment with the two IL-2 types. CD4^+^ and CD8^+^ T cells with a significantly higher frequency of CD183, CD44, and CD54 were observed in T cells treated with FSD13 (Fig. 2c, d). High levels of CXCR3 (CD183) on circulating and tumor-infiltrating CD8^+^ T cells have been implicated in the effective control of advanced melanoma^12^. CD44 and CD54 meditate cell adhesion between immune cells and vascular endothelial cells. Together, these data indicate that T cells activated by FSD13 have a strong capacity to pass through blood vessel endothelium and infiltrate tumor tissue. To verify whether a low dose of FSD13 induced as many Tregs as wild-type IL-2, CD4^+^ T cells were treated with 1640 RIPM (control), FSD13, or wild-type IL-2 for 5 days before FACS analysis. Compared with the frequency of CD4^+^ T cells expressing FoxP3 after treatment with control, only 4.8% of the CD4^+^ T cells treated with FSD13 expressed FoxP3; three times lower than the frequency (17.0%) observed in CD4^+^ T cells treated with wild-type IL-2 (Fig. 3a, b). The data suggest that FSD13 induced fewer Tregs. At the same time, we found that after activation with FSD13, the percentage of CD4^+^ T cells secreting interferon (IFN)-γ was significantly increased (FSD13: 16.9% vs. wild-type IL-2:4.9%, *P* < 0.01) (Fig. 3c, d).

### FSD13 induces stronger NK cell cytotoxicity on tumor cells than wild-type IL-2

**Fig. 4:**
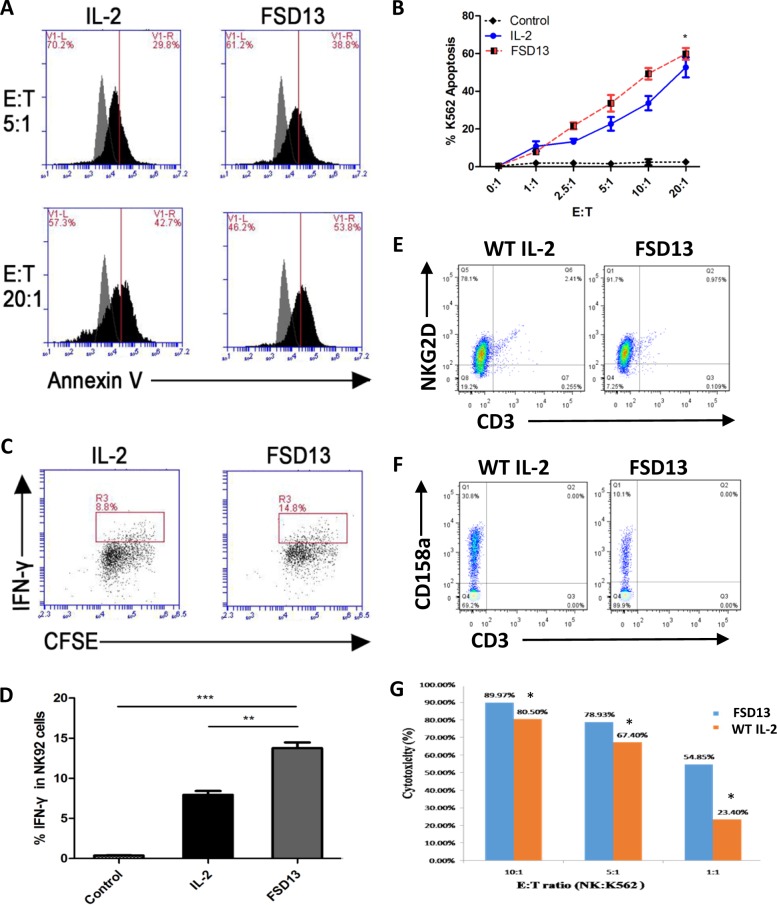
Effect on NK cell-meditated cytotoxicity. NK92 cells were activated by FSD13, IL-2 or PBS (control) for 3 days before co-culture with K562 cells in different ratios (effector : target). **a**, **b** NK92 cells activated by FSD13 increased the frequency of K562 cells undergoing apoptosis. *P < 0.05 (two-way ANOVA). **c**, **d** Compared with IL-2, FSD13 strengthened IFN-γ secretion in NK92 cells. **e** FSD13 increased the frequency of activated primary NK cells. **f** FSD13 lowered the frequency of CD158a on primary NK cells. **g** Primary NK cells activated by FSD13 increased the frequency of K562 cells undergoing apoptosis. *P < 0.05 (Student’s *t*-test). All experiments were conducted three times. Experiments were conducted in five biological replicates (*n* = 5) for each group. Data in (**b**) are represented as mean ± SEM and were collected from three independent experiments

NK92 cells are commonly accepted and recognized as a platform to research NK cell function. Moreover, NK92 cells have been shown to be safe and provide the basis for a potentially beneficial antitumor therapy. The use of NK92 cells has received Food and Drug Administration approval for testing in patients with advanced malignant melanoma and renal cell carcinoma^13–15^. In this NK cell-meditated K562 (human chronic myelogenous leukemia) apoptosis assay, we first used NK92 cells. Before co-culturing them with K562 cells, NK92 cells were activated with wild-type IL-2 or FSD13 for 3 days. RPMI-1640 medium served as control. After the activation of NK92 cells, the cells were uniformly mixed with K562 cells in different ratios and incubated in a CO_2_ incubator containing 5% CO_2_ at 37 °C for 4–6 h. An Annexin-V/7AAD kit was used to determine the percentage of K562 cells undergoing apoptosis induced by NK cell-meditated cytotoxicity. As the ratio of effector cells (NK92 cells) in the co-culture increased, not only did the cytotoxicty of NK92 cells activated by wild-type IL-2 increase, but so did the NK92 cells activated by FSD13. When the effector/target (E/T) ratio was in the 2.5:1 to 10:1 range, FSD13-activated NK92 cells meditated 10% more K562 cells undergoing apoptosis than NK92 cells activated by wild-type IL-2 (Fig. 4a, b). This finding suggests that FSD13 exhibited a stronger effect in strengthening NK cell cytotoxicity against tumor cells than wild-type IL-2. Furthermore, we examined the cellular level of IFN-γ secretion in NK cells induced by wild-type IL-2 and FSD13 using intracellular staining followed by FACS analysis. A significantly increased percentage of IFN-γ secretion was observed in the NK92 cells activated by FSD13 (Fig. 4c, d). By producing IFN-γ, activated NK cells induce CD8^+^ T cells to become cytotoxic T lymphocytes (CTLs) and also help CD4^+^ T cells move toward Th1 response to promote CTL differentiation^16,17^, which creates a more potent immune environment. To further confirm our findings, we isolated human primary NK cells and tested the Killer Activation Receptor NKG2D. FSD13 significantly increased NKG2D-positive NK cells from 78.1% to 91.7% (Fig. 4e), and decreased the Killer Inhibitory Receptor CD158a-positive NK cells from 30.8% to 10.1%. (Fig. 4f). Indeed, primary NK cells with high frequency of NKG2D and low frequency of CD158a stimulated by FSD13 exhibited significantly higher cytotoxicity on K562 tumor cells than those exposed to wild-type IL-2 (Fig. 4g).

### FSD13 presents greater antitumor efficacy than wild-type IL-2 in controlling metastasis

**Fig. 5:**
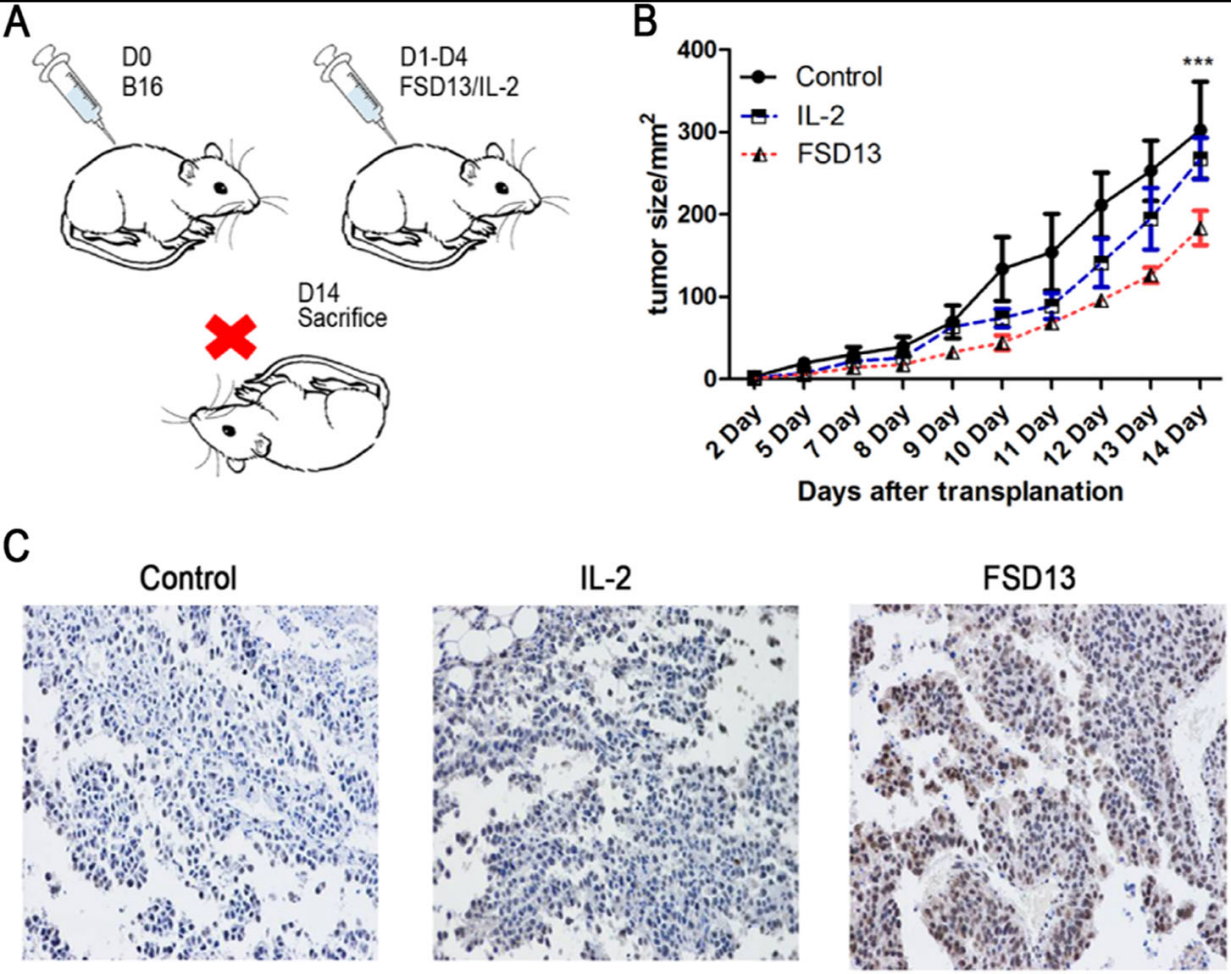
Antitumor effect in B16 transplantation model. Some 2 × 10^5^ B16 cells were injected i.p. into the inguinal region of C57BL/6 mice in each group (n > 5/group) on day 0. Then 20 μg of FSD13 and IL-2 was injected i.p. to control the tumor invasion. **a** Treatment schedule represents the experiments. **b** FSD13 displays a better effect on control tumor growth in situ. **c** Immunohistochemistry staining of CD8+ T cells infiltrating tumor tissue. All experiments were conducted three times. Experiments were conducted in six biological replicates (*n* = 6) for each group. Data are represented as mean ± SEM and were collected from three independent experiments. ***P < 0.01 (Student’s *t*-test)

Malignant melanoma, which develops from a neoplastic transformation of melanocytes, is the most aggressive form of skin cancer, and the number of cases has increased 2.8% annually since 1981 in the United States alone^18^. What makes the lung metastatic model so meaningful is that wild-type IL-2 is frequently used in clinical therapy to prevent tumor cells from metastasizing and to accelerate the regression of melanoma. B16 cells (2 × 10^5^) were injected intravenously into C57BL/6 mice in each group. Following injection, wild-type IL-2, FSD13, or phosphate-buffered saline (PBS) (as control) were continuously used to treat the mice in different groups on days 1–4. Mice were killed on day 21 (Fig. 5a). Melanoma metastatic nodules on the lungs in each group were carefully counted and the largest nodule was measured to represent the malignancy of the tumor. Compared with the control group, the numbers of metastatic nodules in both the wild-type IL-2 (mean: 65) and the FSD13 (mean: 10) groups were significantly less (Fig. 5b, c) than in the control group. Furthermore, FSD13 exhibited better antitumor efficacy than wild-type IL-2 (*P* < 0.05). The biggest nodule on the lungs of mice in the FSD13 group was significantly smaller than that found in the control (*P* < 0.01) and wild-type IL-2 (*P* < 0.05) groups, which also suggested a stronger antitumor capability (Fig. 5d). During dissection of the mice, the investigator found that tumor cells had spread widely via blood circulation and adjacent invasion to areas such as the chest wall and right kidney in the control and wild-type IL-2 groups, but such widely spaced nodules were seldom found in the FSD13 group. These findings indicate that FSD13 has better anti-metastatic ability than wild-type IL-2.

### FSD13 more effectively undermines growth of melanoma in situ

**Fig. 6:**
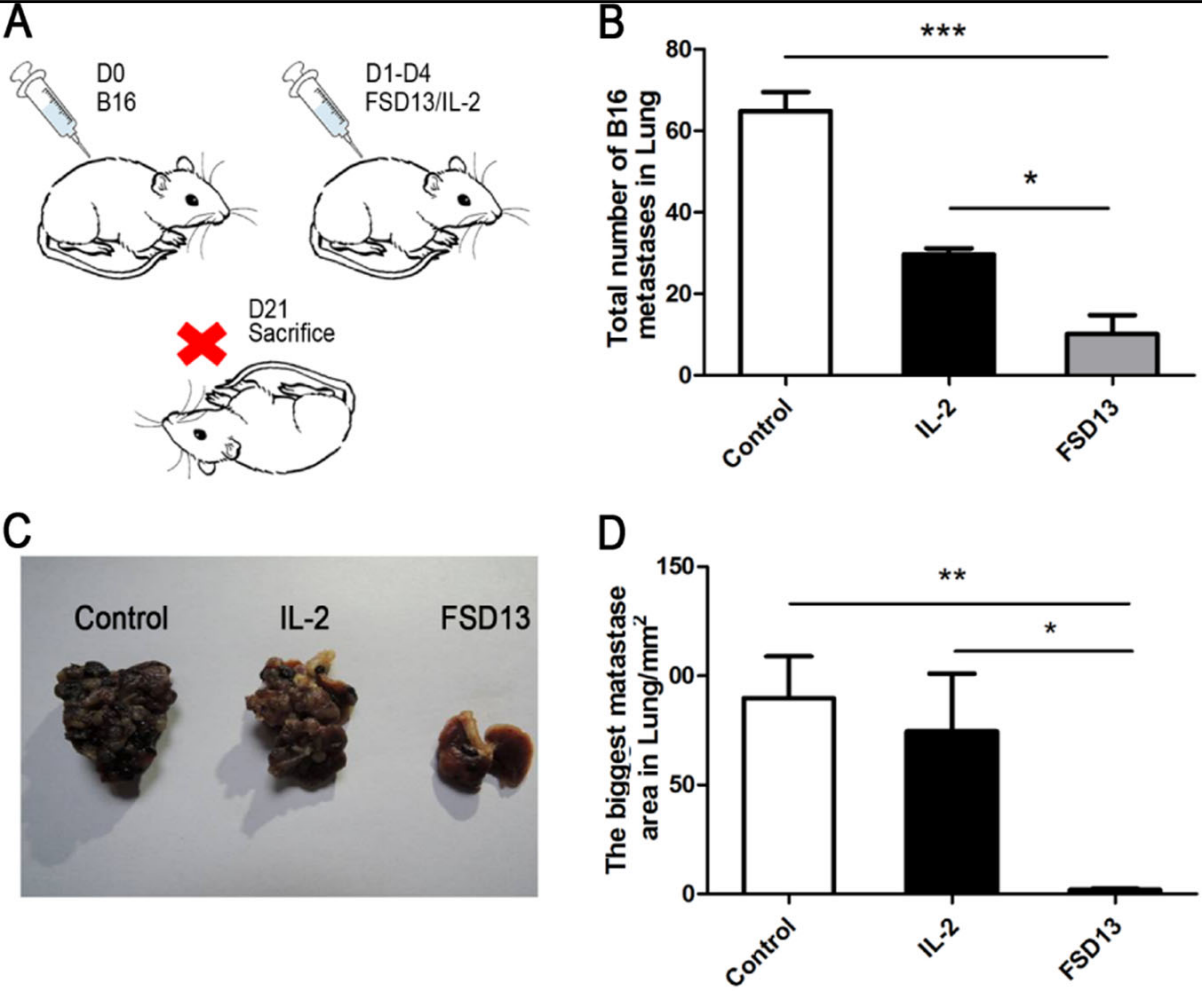
Antitumor effect in a B16 lung metastases model. Some 2 × 10^5^ B16 cells were injected i.v. into C57BL/6 mice in each group (*n* > 5/group) on day 0. Then 20 μg of FSD13 and IL-2 was injected i.p. to treat mice bearing tumor cells. PBS served as control. After mice were killed, the metastasic nodules on lung were counted and the biggest nodule was measured. **a** Procedure represents the experiments. **b**, **c**, **d** FSD13 exhibited stronger antitumor effects than IL-2. All experiments were repeated three times. Experiments were conducted in more than six biological replicates (*n* = 6) for each group. Data were analyzed by Student’s *t*-test and represented as mean ± SEM, and were collected from three independent experiments. *P < 0.05, **P < 0.01, ***P < 0.001

Before melanoma cells metastasize, they extend into the adjacent epidermis^10^ and grow, compressing adjacent tissues. The melanoma in situ serves as the original source of the tumor cells. If it can be suppressed, the risk of formation of melanoma metastases may be decreased. In our transplantation model, 2 × 10^5^ B16 cells were subcutaneously injected into the inguinal region of mice in three groups (*n* > 5/group) on day 0. The same dose of wild-type IL-2, FSD13, or PBS (control) were inoculated i.p. on days 1–4 into mice in different groups, continuously. Mice were killed on day 14 (Fig. 6a). Within the first week, both wild-type IL-2 and FSD13 showed clear antitumor effects. From day 8 we observed that melanoma on the mice treated with wild-type IL-2 grew faster at an accelerating pace, reaching 260 mm^2^ on average on day 14, which was only 40 mm^2^ smaller than the size of the melanoma measured in the PBS group. Compared with the other groups, the melanoma size in mice in the FSD13 group was significantly smaller (*P* < 0.05), which suggests that FSD13 more effectively undermines the growth of melanoma in situ (Fig. 6b). During the dissection of the mice, the investigator found that the boundaries between the normal and tumor tissues were unclear in the control and wild-type IL-2 groups. Tumor had invaded into muscle and was difficult to separate in the two groups. In contrast, tumor tissue in the FSD13 group mice had clear boundaries between normal and tumor tissue, indicating melanoma in the FSD13 group mice remained stalled at an early stage of development. In addition, immunohistochemical staining of mice from the FSD13 group showed greater CD8^+^ T-cell infiltration in tumor tissue than in the other groups (Fig. 6c).

### FSD13 leads to less organ toxicity than wild-type IL-2

**Fig. 7:**
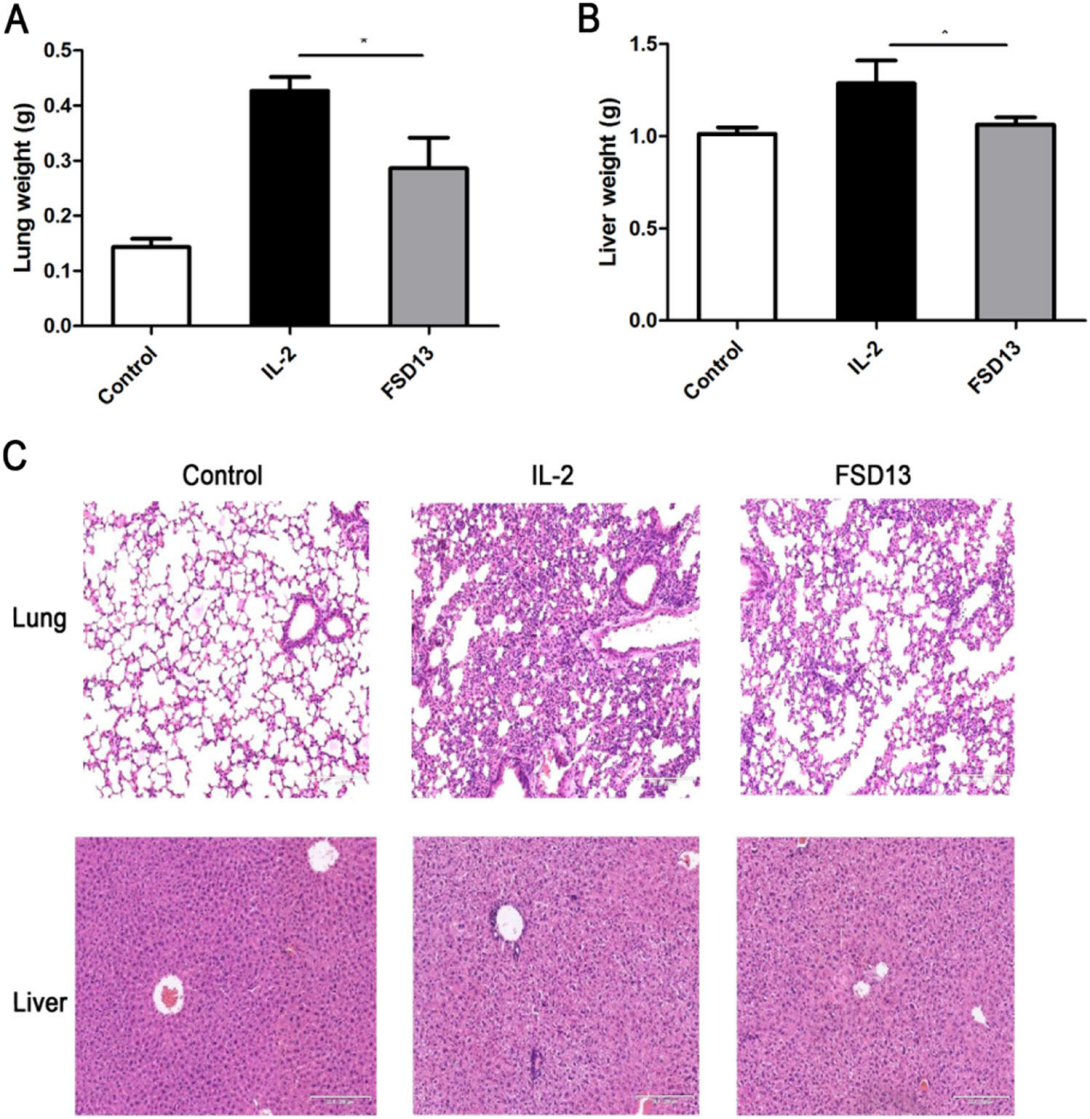
Effect on organic toxicity. 80μg FSD13 or IL-2 was injected i.p. into C57BL/6 mice in each group (*n* = 6/ group) for five days, before mice were killed. Weight of lung and liver was determined. Four percent paraformaldehyde was used to fixed mice organ for further H&E staining. **a** FSD13 exhibited less organic toxicity than IL-2. **b** H&E staining showed FSD13 caused less lung edema and liver cell damage. All experiments were conducted three times. Experiments were conducted in six biological replicates (*n* = 6) for each group. Data were represented as mean ± SEM and were collected from three independent experiments. **P < 0.01 (Student’s *t*-test), *P < 0.05 (Student’s *t*-test)

Organ toxicity caused by wild-type IL-2 mainly results from increased vascular permeability, which in turn leads to VLS^19^. Mice were separated randomly into three groups (*n* = 6/group). We used 80 μg wild-type IL-2 or FSD13 (PBS as control), which was four times higher than the dose used to treat mice in the lung metastasis and B16 cell transplantation model, to induce organ-specific toxicity in mice and simulate the process of increasing vascular permeability in vivo. Lung and liver are frequently found to be damaged by IL-2 based immunotherapy. In our induced organ toxicity model, mice were killed after different types of IL-2 treatments for 5 days, and the lungs and livers weighed. Compared with the mice treated with PBS, a significant weight increase in the lung (*P* < 0.05) and liver (*P* < 0.05) was observed in wild-type IL-2 treated mice, whereas such an increase was not seen in FSD13-treated mice (Fig. 7a). In addition, analysis showed marked histological changes in lung and liver, revealing severe lung edema and liver cell damage in mice injected with wild-type IL-2 compared with mice in the control group (Fig. 7b). There were no dramatic morphological alterations in the lungs and livers collected from mice in the FSD13 group upon histological examination. These data strongly suggest FSD13 induces less toxicity than wild-type IL-2.

**Fig. 8:**
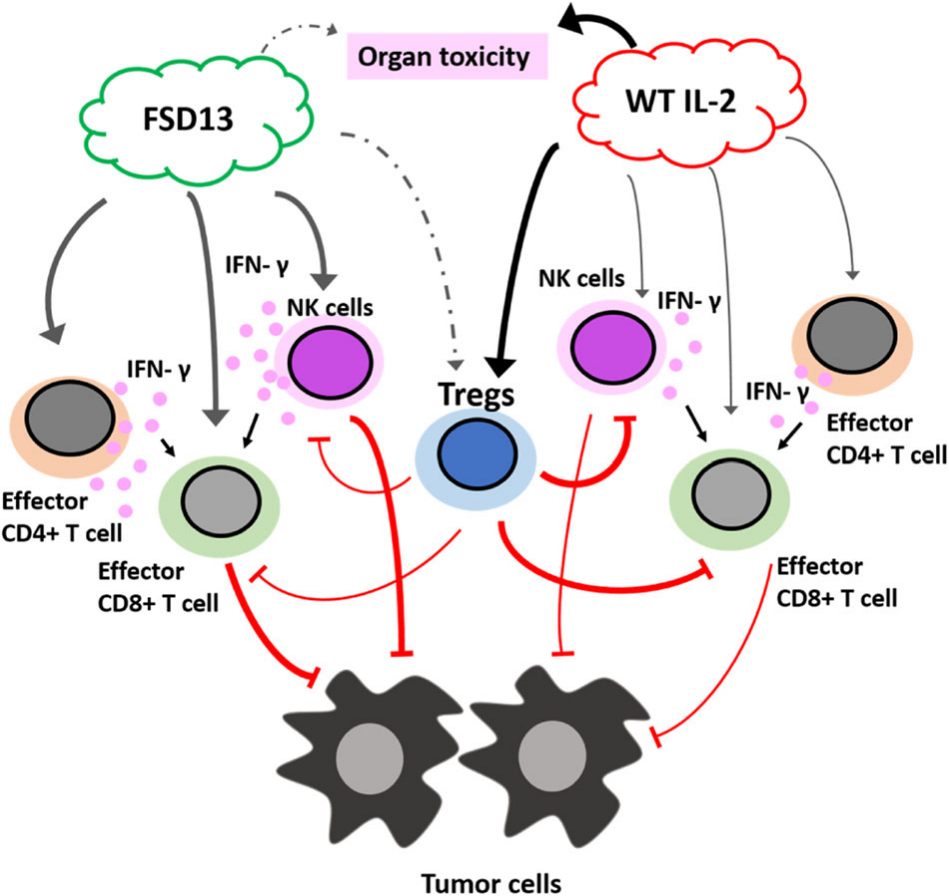
The differential effects of FSD13 and wild-type IL-2 on antitumor efficacy and organ toxicity. FSD13 exhibits superior effects than wild-type IL-2 on activation of effector CD4^+^ T cells, CD8^+^ T cells, and NK cells. FSD13 has a greater ability than wild-type IL-2 in inducing IFN-γ production, which activates CD8^+^ T cells. Treg cells limit the activity of CD8^+^ T cells and NK cells in killing tumor cells. FSD13 activates less Tregs than wild-type IL-2. Moreover, FSD13 is safer than wild-type IL-2 in the respect of inducing lesser organ toxicity

Thus, we demonstrated the differential effects of FSD13 and wild-type IL-2 on antitumor efficacy and organ toxicity (Fig. 8). FSD13 has superior effects than wild-type IL-2 on activation of effector T cells and NK cells (Fig. 8). FSD13 activates less Tregs than wild-type IL-2 (Fig. 8). Moreover, FSD13 is safer than wild-type IL-2 in the respect of inducing lesser organ toxicity (Fig. 8).

## Discussion

Our study demonstrates that FSD13 increases the cytokine’s stimulation of CD8^+^ T cells and NK cells with little effect on Treg cells. Furthermore, our data from tumor cell metastases and transplantation models in mice show that FSD13 has more tumor suppressive effects than wild-type IL-2. We assume the reasons for this difference are that FSD13 decreased the number of Tregs during the proliferation of CD4+ T cells, and that FSD13 can activate T and NK cells much faster than wild-type IL-2. From our data, compared with the administration of wild-type IL-2, after FSD13 treatment, both T and NK cells proliferated much faster, especially between days 1 and 3. This period showed the most obvious improvement in the efficacy of FSD13. Furthermore, FSD13 promoted NK cell-meditated tumor cell apoptosis. As NK cells have no major histocompatibility complex (MHC) restriction, NK cells can kill a wide range of tumor cells, demonstrating that NK cells play an important role in suppressing tumor invasion. In addition, FSD13 encourages antitumor immune cells to infiltrate tumor tissues, and thus helps the immune system eliminate tumor invasion more effectively. In comparison, administration of wild-type IL-2 in a clinical setting not only induces Treg proliferation, but also results in a weak immune response^20^, as well as severe risk of VLS. Therefore, we believe that FSD13 has great potential for clinical application to treat various cancers since it avoids the main disadvantages of IL-2 based immunotherapy.

An increasing number of studies have found that wild-type IL-2 has a dominant role in immune suppression rather than in immune stimulation as originally thought^21,22^. Two groups have reported that in cancer patients the number of Tregs increased after IL-2 treatment^23^. Therefore, a new IL-2 mutant that reduces Treg proliferation would greatly improve IL-2 based immunotherapy and could bring significant benefits to patients suffering from metastatic cancer. Using in vitro evolution, Levin et al.^24^ eliminated IL-2’s requirement to bind to CD25 and generated an IL-2 superkine with increased affinity toward the IL-2R β-subunit. Our FSD13 is equal or better to that of the IL-2 superkine because it does not induce edema in the lung or damage the liver, at least in an animal model.

In clinical practice, IL-2 administration to patients with metastatic renal cell carcinoma or melanoma achieved a relatively low rate of remission, and toxicity was severe^3^. Our study confirms the recent consensus that selective inhibition of IL-2-mediated enhancement of Tregs may improve the therapeutic effectiveness of IL-2^21,24^. We believe that using FSD13 to treat cancer patients will accomplish at least the same remission rate as IL-2 but with much lower risk of any significant side effects.

Recent studies demonstrate that a subset of CD4^+^ T cells, referred to as CD4^+^CD25^hi^ FoxP3^+^ naturally occurring Tregs, may accumulate in the tumor environment and suppress tumor-specific T-cell responses, thereby hindering tumor rejection^25–27^. CD4^+^CD25^+^ FoxP3^+^ T cells do not suppress the activation of CD4^+^ T cells until the response cells produce IL-2^28,29^. This finding indicates that IL-2 is necessary for Treg function. Fortunately, the capacity to induce Tregs massive proliferation does not occur after FSD13 administration, as demonstrated by testing the frequency of CD25^+^FoxP3^+^ T cells in CD4^+^ T cells purified from human peripheral blood cells using FACS.

It is critical to identify the site on IL-2 responsible for Treg induction, because such identification enabled us to eliminate the tolerance-inducing aspect of IL-2. On the basis of our results, the FSD13 we identified essentially abolishes the issue of Treg-induced immune tolerance, while retaining the cytokine antitumor ability. FSD13 has shown a capacity to support IL-2 dependent NK92 cell proliferation and stimulate the activation of CD8^+^ T cells. From these studies, it appears that the two major divergent functions of wild-type IL-2 (stimulating immune function and inducing immune tolerance) can be functionally separated and altered individually by lysine amino acid substitutions.

In summary, our data indicates that FSD13 produced by a selective amino acid substitution can effectively eliminate immune tolerance without affecting the cytokine functionality on CD8^+^ T and NK cells. Therefore, selective inhibition of IL-2-mediated enhancement of Treg proliferation may improve the therapeutic effectiveness of IL-2-based immunotherapy in the future.

## Materials and methods

### Mice

All specific pathogen free (SPF) mice used were 4–6 weeks old, and purchased from the Shanghai Laboratory Animal Center. All animal experiments followed the guidelines of the International Council for Laboratory Animal Science and were performed under the norms of the Institutional Animal Care and Use Committee. Mice were randomly assigned into different experimental groups. One investigator performed the group allocation and treatment, and the other investigator, who was blinded to the group allocation and treatment, conducted the endpoint analyses.

### Generation of IL-2 clone and analog

The IL-2 was amplified by reverse-transcription PCR with the 5′ and 3′ primers 5′-GGTAAAGCGGCCGAGCACCTACTTCAAGTTCTACA-3′ and 5′-TCATGCGGCCGCTCAAGTTAGTGTTGAGATGATGCT-3′, respectively, to append a NotI restriction site and codons for a polypeptide linker to the 5′-end of the IL-2 cDNA and a stop codon at the 3′-end. Following gel purification of NotI restriction endonuclease digestion of the PCR product and pcDNA3.1-his DNA, the vector fragment was dephosphorylated by using Shrimp Alkaline Phosphatase and the PCR fragment was ligated into pcDNA3.1-his, which resulted in the expression vector pcDNA3.1-his-hIL2. IL-2 analog (FSD13) was made by P85R site-directed mutation.

### Expression and purification of IL-2 mutant (FSD13)

His-tagged native IL-2 and IL-2 analogs (16 kDa) were expressed in CHO cells according to the manufacturer’s protocol and the secreted proteins were purified using Ni-NTA Superflow Columns (Qiagen). The size of the purified proteins were verified by SDS-polyacrylamide gel electrophoresis and transferred to a membrane and probe with anti-IL-2 antibody. Their concentrations were measured by SpectraMax.

### Primary cells, cell lines, cell line authentication, and culture conditions

CD4^+^, CD8^+^T, and primary NK cells were separated from PBMC by magnetic separation. Human whole blood samples came from the blood donation station of Changhai Hospital’s blood donation center. Cells were cultured in PRMI1640 (Hyclone) supplemented with 10% fetal bovine serum (FBS) (Gibco, USA). NK92 (ATCC® CRL-2407™) and K562 cells (ATCC® CCL-243™) were cultured in PRMI1640 (Hyclone) with 10% FBS (Gibco, USA). B16-F0 (ATCC® CRL-6322™) cells were maintained in Dulbecco’s modified Eagle’s medium (Hyclone) with 10% FBS (Gibco,US). All cells were cultured under a humidified 5% CO_2_ atmosphere at 37 °C for further analysis. All cell lines were obtained from ATCC and used within low passages (< 6). ATCC claims that has comprehensively performed authentication and quality-control tests on all distribution lots of cell lines. Obtaining and using low-passage cell lines from ATCC is a sure way to work and publish with confidence. We have included the designation, the ATCC catalog number, and the passage numbers under which experiments were conducted in the materials and methods, e.g., NK92 (ATCC® CRL-2407™). In addition, we checked cell morphology by microscope weekly to monitor potential abnormal morphology changes, and biweekly detected mycoplasma infection using the Hoechst 33258 staining. Fluorescent Hoechst 33258 staining reveals mycoplasma infections through their characteristic patterns of extracellular particulate or filamentous fluorescence at ×500 magnification. We have included the above statement in the Material and Methods.

### Proliferation assay

Purified CD4^+^T, CD8^+^T and NK92 cells were collected and stained with CFSE according to the CFSE staining protocol before being cultured in a 96-well plate (1 × 10^5^/well) for 1–7 days. A certain concentration of wild-type IL-2 or FSD13 was added to stimulate the T cells to proliferate before the cells were analyzed by flow cytometry on days 1, 3, 5, and 7. Samples were analyzed using a Cytometers Accuri C6 (BD, USA).

### NK cells cytotoxicity assay

Before being co-cultured with K562, primary NK cells and NK92 cells were activated by wild-type IL-2 or FSD13 for 3 days. For the analysis of the antitumor efficacy of different IL-2 activated NK92 or primary NK cells, NK92 or primary NK cells were uniformly mixed with K562 in a series of different E/T ratios for 4–6 h. An Annexin-V/7-ADD kit (BD, USA) was used to determine the percentage of K562 cells undergoing apoptosis under the recommended protocol. Samples were analyzed using a Cytometers Accuri C6 (BD, USA).

### Flow cytometry and magnetic cell separation

For flow cytometry assay, samples were suspended and adjusted to standard concentrations according to specifications. All fluochrome-conjugated Abs, unless specifically stated, were purchased from eBiosicience. The PE anti-human CD69, APC-anti-human FoxP3, and human Foxp3 staining buffer sets were purchased from BD (USA). For magnetic cell separation, PBMCs were separated from whole blood using Lymphoreo (Fresenius Kabi Norge AS, Norway). Then the proper volume (10 μl per 10^6^ cells) of anti-CD4 or anti-CD8 microbeads (Miltenyi, De) was added to the cell suspension and allowed to react on ice for 15 min before the cells went through a magnetic separator. The magnetic separator was washed three times to thoroughly separate CD4^−^ or CD8^−^ cells from CD4^+^ or CD8^+^ cells. The positive cells were then collected from the magnetic separator.

### Lung metastasis model

To establish the lung metastasis model, mice were divided into three groups: wild-type IL-2, FSD13, and PBS. Each group consisted of six mice. Some 2 × 10^5^ B16 cells were injected into each C57BL/6 mouse via the tail vein on day 0. After injection, on days 1–4, 20 μg of wild-type IL-2 diluted in 200 μl PBS, 20 μg FSD13 diluted in 200 μl PBS or 200 μl PBS were injected i.p into each C57BL/6 mouse in each group. All mice were killed on day 21. Tumor nodules on the mice’s lungs were counted and the largest nodules were measured using an electronic Vernier caliper.

### Tumor cell subcutaneous transplantation model

Some 2 × 10^5^ B16 cells were injected into each C57BL/6 mouse subcutaneously on day 0 in preset groups (wild type, FSD13, and PBS). After injection, 20 μg of clinical wild-type IL-2 diluted in 200 μl PBS, 20 μg FSD13 diluted in 200 μl PBS or 200 μl PBS were injected i.p into different groups of C57BL/6 mice on days 1–4. Hair on the injection area was cleaned to make it easier to observe tumor growth. A Vernier caliper was used to measure the size of the melanoma nodules. Measurements began when a black dot was visible on the skin. Mice were killed on day 21 or when the skin covering a tumor began to ulcerate. The tumor was removed and embedded in 4% paraformaldehyde for further immunohistochemistry analysis.

### Induced toxicity model

Some 80 μg of wild-type IL-2 diluted in 300 μl PBS, 80 μg FSD13 diluted in 300 μl PBS, or 300 μl PBS (control) were injected i.p into a C57BL/6 mouse in the IL-2, FSD13, and PBS groups (*n* = 6/group) on days 1–5. Mice were killed on day 6. Lung and liver were removed and weighed before being transferred into 4% paraformaldehyde for fixation. Paraffin-embedded sections were stained with hematoxylin and eosin staining.

### Statistical analysis

Data are expressed as means ± SEM. A statistical analysis was conducted using SPSS (18.0, IBM, USA) and Graph Pad Prism 5.0. Significance was set at 5% after a difference was demonstrated using Student’s *t*-test and analysis of variance (ANOVA). The tumor curve data were analyzed by two-way ANOVA with Bonferroni correction.
